# Effect of a Telecare Case Management Program for Older Adults Who Are Homebound During the COVID-19 Pandemic: A Pilot Randomized Clinical Trial

**DOI:** 10.1001/jamanetworkopen.2021.23453

**Published:** 2021-09-09

**Authors:** Arkers Kwan Ching Wong, Frances Kam Yuet Wong, Karen Kit Sum Chow, Siu Man Wong, Paul Hong Lee

**Affiliations:** 1 School of Nursing, The Hong Kong Polytechnic University, Hung Hom, Hong Kong; 2 The Hong Kong Lutheran Social Service, Homantin, Hong Kong; 3 Department of Health Sciences, University of Leicester, Leicester, United Kingdom

## Abstract

**Importance:**

Older adults who are homebound can be difficult to reach owing to their functional limitations and social distancing during the COVID-19 pandemic, leaving their health needs unrecognized at an earlier stage.

**Objective:**

To determine the effectiveness of a telecare case management program for older adults who are homebound during the COVID-19 pandemic.

**Design, Setting, and Participants:**

This randomized clinical trial was conducted among 68 older adults in Hong Kong from May 21 to July 20, 2020, with a last follow-up date of October 20, 2020. Inclusion criteria were being 60 years or older, owning a smartphone, and going outside less than once a week in the previous 6 months.

**Interventions:**

Participants in the telecare group received weekly case management from a nurse supported by a social service team via telephone call and weekly video messages covering self-care topics delivered via smartphone for 3 months. Participants in the control group received monthly social telephone calls.

**Main Outcomes and Measures:**

The primary outcome was the change in general self-efficacy from before the intervention to after the intervention at 3 months. Self-efficacy was measured by the Chinese version of the 10-item, 4-point General Self-efficacy Scale, with higher scores representing higher self-efficacy levels. Analysis was performed on an intention-to-treat basis.

**Results:**

A total of 68 participants who fulfilled the criteria were enrolled (34 in the control group and 34 in the intervention group; 56 [82.4%] were women; and mean [SD] age, 71.8 [6.1] years). At 3 months, there was no statistical difference in self-efficacy between the telecare group and the control group. Scores for self-efficacy improved in both groups (β = 1.68; 95% CI, −0.68 to 4.03; *P* = .16). No significant differences were found in basic and instrumental activities of daily living, depression, and use of health care services. However, the telecare group showed statistically significant interactions of group and time effects on medication adherence (β = −8.30; 95% CI, −13.14 to −3.47; *P* = .001) and quality of life (physical component score: β = 4.99; 95% CI, 0.29-9.69; *P* = .04).

**Conclusions and Relevance:**

In this randomized clinical trial, participants who received the telecare program were statistically no different from the control group with respect to changes in self-efficacy, although scores in both groups improved. After the intervention, the telecare group had better medication adherence and quality of life than the control group, although the small sample size may limit generalizability. A large-scale study is needed to confirm these results.

**Trial Registration:**

ClinicalTrials.gov Identifier: NCT04304989

## Key Points

**Question:**

Can a telecare case management program delivered by a nurse case manager supported by a health-social team improve self-efficacy, health-related measures, and health care service utilization outcomes among older adults who are homebound?

**Findings:**

In this randomized clinical trial with 68 participants, there was no statistical difference in self-efficacy between the telecare group and control group at 3 months according to the Chinese version of the 10-item, 4-point General Self-efficacy Scale. Scores for self-efficacy improved in both groups over time.

**Meaning:**

While the intervention did not increase self-efficacy, the findings suggest that telecare case management may increase quality of life and rates of medication adherence among older adults who are homebound.

## Teaser

This randomized clinical trial evaluates the effectiveness of a telecare case management program at allowing older adults who are homebound in Hong Kong to stay in the community with optimal well-being during the COVID-19 pandemic.

## Introduction

The number of older adults who are homebound is growing substantially owing to a global aging population and advanced health care technology. A current report from the United States shows that approximately 3 million community-dwelling older adults are in a chronic homebound state,^1^ that is, confined to their home and normally unable to go outdoors more than once per week because of physical and functional impairments.^2^ Older adults who are homebound encounter a range of physical ailments, such as chronic pain and muscle weakness, which prevent them from leaving their homes.^3,4^ It is therefore unsurprising that older adults who are homebound have a higher prevalence of polypharmacy and greater health care service utilization than their counterparts who are not homebound.^5^

Home-based primary care services are promoted among the older population to improve their access to health care and their ability to remain safely in their own homes without being institutionalized.^6^ Home-based primary care is a multidisciplinary support model involving services ranging from assistance with basic daily living to tertiary-level health care for those who have difficulty accessing office-based primary care.^7^ A systematic review has demonstrated that this model could improve quality of life (QOL) and satisfaction levels and reduce depression levels and disability among older adults who are homebound.^8^ However, despite the effectiveness of these home-based services, studies indicate that additional time and resources are required for health care professionals to travel to homes, diverting resources from hospital settings and possibly affecting quality of care in acute settings.^9,10^ Physical home visits also increase risks of infectious disease transmission, which is a particular concern during the current COVID-19 pandemic. Home-based health care services are limited during this pandemic period.^11^ An alternative home-based care delivery model is needed that can be sustained throughout and beyond the pandemic.

Telecare refers to the use of electronic modalities, such as smartphones, to support long-distance, virtual, face-to-face encounters between health care professionals and their patients.^12^ Telecare particularly benefits older adults who are homebound and have limited access to customary health care services owing to physical disability^13^ and face a worsened situation during the pandemic. However, the utilization rate of these telehealth programs among older adults who are homebound was low, with reported challenges that included technical compatibility issues and the high cost of installation and maintenance of home health monitoring services.^14^

Older adults who are homebound often rely on other people to undertake activities that require leaving home, such as accessing health care services.^5^ Compounded by a decrease in community engagement and functional limitations,^15^ these individuals have a greatly compromised sense of self-efficacy,^16^ which may hamper their ability to maintain healthy living at home.^15^ Self-efficacy is frequently regarded as an indicator of a person’s initiation of and motivation for engaging in self-care practice, and previous studies have reported that persons with higher self-efficacy have a better QOL and use fewer hospital services.^17^ The introduction of telecare delivery of some health care services can make bolstering the self-efficacy level of older adults who are homebound plausible. By using telecare, health care professionals can readily offer health information that is relevant to older adults’ individual needs and co-design their self-directed goals and action plan for goal attainment without requiring them to leave home. Previous telecare studies have shown promising results in improving the self-efficacy level of patients with chronic diseases.^18,19^ Older adults who are homebound have been identified as a population with low levels of self-care self-efficacy.^20^ This research therefore evaluated a telecare program delivered by a nurse case manager with the integrated efforts of a health-social team.

The study aims were to test the effectiveness of a telecare case management program on self-efficacy, health-related outcomes (activities of daily living [ADLs], instrumental ADLs [IADLs], and medication adherence), perceived well-being (QOL, depression), and use of health care services (outpatient clinic visits, private general practitioner visits, emergency department visits, and hospital admissions).

## Methods

For this pilot randomized clinical trial, ethical approval was obtained from the Human Subjects Ethics Subcommittee of The Hong Kong Polytechnic University. All participants were provided with a full explanation of the study and signed their informed consent prior to baseline data collection. The study followed the Consolidated Standards of Reporting Trials (CONSORT) reporting guideline (the full trial protocol appears in Supplement 1).

### Design and Setting

This study adopted a single-blind, 2-group randomized clinical trial design in which the data collector was blinded but the participants and health care professionals were not. Recruitment took place in 5 Hong Kong community centers.

### Participants, Recruitment Strategy, and Randomization

From May 21 to July 20, 2020, the managers in each of the 5 community centers referred 99 individuals who were homebound (defined as going outdoors less than once a week in the previous 6 months)^21^ and residing within the community service area. Of these individuals, 68 were eligible. They completed the baseline data collection and were enrolled and randomized into groups (Table 1). Inclusion criteria for this study were being 60 years or older and using a smartphone. Exclusion criteria were receiving a diagnosis of dementia; inability to hear, see, or communicate; being confined to bed; having an active psychiatric illness with hospitalization within the previous 6 months; residing in an area with no internet coverage; and having been engaged in other telecare programs.

**Table 1.** Demographic Characteristics of Participants

Characteristic	No. (%) or No./total No. (%)		
	Total (N = 68)	Intervention group (n = 34)	Control group (n = 34)
Sex			
Male	12 (17.6)	6 (17.6)	6 (17.6)
Female	56 (82.4)	28 (82.4)	28 (82.4)
Age, y			
Mean (SD)	71.8 (6.1)	72.2 (5.9)	71.3 6.1
Median (range)	71 (61-85)	71 (62-85)	71 (61-84)
Marital status			
Single	6 (8.8)	2 (5.9)	4 (11.8)
Married	34 (50.0)	17 (50.0)	17 (50.0)
Divorced	5 (7.4)	1 (2.9)	4 (11.8)
Widowed	23 (33.8)	14 (41.2)	9 (26.5)
Educational level			
No formal education	5 (7.4)	1 (2.9)	4 (11.8)
Primary	24 (35.3)	12 (35.3)	12 (35.3)
Secondary	37 (54.4)	21 (61.8)	16 (47.1)
Tertiary or above	2 (2.9)	0	2 (5.9)
Occupation			
Full-time	0	0	0
Part-time	1/67 (1.5)	1/33 (3.0)	0
Unemployed	2/67 (3.0)	1/33 (3.0)	1/34 (2.9)
Retired	64/67 (95.5)	31/33 (93.9)	33/34 (97.1)
Living area			
Apartment	68 (100)	34 (100)	34 (100)
A room	0	0	0
A bed	0	0	0
Living with			
Alone	21 (30.9)	9 (26.5)	12 (35.3)
Spouse	17 (25.0)	10 (29.4)	7 (20.6)
Child	30 (44.1)	15 (44.1)	15 (44.1)
Financial status			
More than sufficient	7 (10.3)	4 (11.8)	3 (8.8)
Barely sufficient	57 (83.8)	29 (85.3)	28 (82.4)
Not sufficient	4 (5.9)	1 (2.9)	3 (8.8)
Far from sufficient	0	0	0
Financial support			
From salary			
No	67 (98.5)	33 (97.1)	34 (100)
Yes	1 (1.5)	1 (2.9)	0
From relative			
No	32 (47.1)	15 (44.1)	17 (50.0)
Yes	36 (52.9)	19 (55.9)	17 (50.0)
From savings			
No	42 (61.8)	23 (67.6)	19 (55.9)
Yes	26 (38.2)	11 (32.4)	15 (44.1)
From pension			
No	60 (88.2)	30 (88.2)	30 (88.2)
Yes	8 (11.8)	4 (11.8)	4 (11.8)
CSSA			
No	64 (94.1)	32 (94.1)	32 (94.1)
Yes	4 (5.9)	2 (5.9)	2 (5.9)
Old age allowance (HKD 1235/mo)			
No	33 (48.5)	19 (55.9)	14 (41.2)
Yes	35 (51.5)	15 (44.1)	20 (58.8)
Normal disability allowance (HKD 1450/mo)			
No	66 (97.1)	32 (94.1)	34 (100)
Yes	2 (2.9)	2 (5.9)	0
High disability allowance (HKD 3160/mo)			
No	68 (100)	34 (100)	34 (100)
Yes	0	0	0
Caregiver			
Other than self	68 (100)	34 (100)	34 (100)
Spouse	23 (33.8)	11 (32.4)	12 (35.3)
Sibling	5 (7.4)	2 (5.9)	3 (8.8)
Child	29 (42.6)	16 (47.1)	13 (38.2)
Son-in-law or daughter-in-law	2 (2.9)	1 (2.9)	1 (2.9)
Friend	6 (8.8)	3 (8.8)	3 (8.8)
Neighbor	1 (1.5)	0	1 (2.9)
Center volunteer	7 (10.3)	3 (8.8)	4 (11.8)
Domestic helper	2 (2.9)	1 (2.9)	1 (2.9)

Abbreviation: CSSA, comprehensive social security allowance.

A research team member (A.K.C.W.) who was not involved in participant recruitment compiled a random assignment schedule according to Research Randomizer software^22^ and placed it in a sealed envelope. Once the trained data collectors had collected the participants’ baseline data in the community centers, they informed a designated team member who was unaware of the participants’ identity. The team member then opened the envelope and revealed the group assignment sequentially based on the computer number (1 = telecare group; 2 = control group).

### Interventions

#### Telecare Group

The participants in the telecare group were assigned to a nurse for the duration of this 3-month program. The nurse, functioning as a case manager (NCM), conducted an initial assessment of the participant using the Omaha system via telephone. The Omaha system is a comprehensive assessment tool used widely to help nurses to identify client needs and problems in environmental, psychosocial, physiological, and health-related behavior.^23^ Working as a team, the NCM and social worker classified the problems listed in the Omaha system into health, social, and health-social partnership focuses. The NCM was primarily responsible for health-focused problems and worked with the social worker to address the other problems. The NCM and the social worker also had monthly case conferences to review the participants’ progress and co-designed, modified, or adjusted their treatment plans accordingly.

Based on the first assessment result, the NCM empowered the participant to design realistic, achievable individual goals and plans and proactively resolved the barriers to implementation. The NCM would also send weekly, individual-specific videos of tips and reminders through WhatsApp via smartphone to the participants to acquaint them with the skills required to perform self-care activities in health maintenance. The topics included, but were not limited to, self-monitoring of a health condition, medication information, health-promoting activities, self-care practices, and appropriate sources of help in the community. All videos provided to the participants were valid and originated from reliable sources, such as the Hospital Authority and the Department of Health.

#### Control Group

Monthly social telephone calls were made to the participants in the control group by a research assistant who was not involved in data collection. Topics such as, “What have you done today?” and “What is your favorite TV program?” were discussed during the social calls, with the aim to minimize possible social support felt by the older adults during the telephone call.

### Outcome Measures

Data were collected at 2 time points: before the intervention at screening (T1) and after the intervention within 1 week after the completion of the 3-month program (T2). The data were collected via telephone by the research assistant, with a last follow-up date of October 20, 2020.

#### Primary Outcome

Self-efficacy was the primary outcome of this study, measured by the Chinese version of the 10-item General Self-efficacy Scale.^24^ Scores were measured on a 4-point Likert scale, with higher scores representing higher self-efficacy levels. The scale was validated in older Chinese adults with a Cronbach α = 0.89.^24^

#### Secondary Outcomes

##### Health-Related Outcomes

Activities of daily living were measured by the 10-item Chinese version of the Barthel Index.^25^ The total scores ranged from 0 to 100, with higher scores indicating greater ability to perform ADLs, such as toileting and grooming. The scale was proven as a valid and reliable tool in a local study.^25^

The 9-item Chinese version of the Lawton Instrumental Activities of Daily Living scale was used to measure participants’ IADLs.^26^ The scale has shown good internal consistency and interrater reliability. Scores were measured on a scale of 0 to 3, with 0 indicating that the individual was incapable of performing an IADL and 3 indicating that the individual was capable of performing an IADL independently.

Medication adherence was measured by the 12-item Adherence to Refills and Medication Scale.^27^ Total scores ranged from 12 to 48, with lower scores representing better medication adherence. The Adherence to Refills and Medication Scale has demonstrated high validity in identifying medication adherence issues in older adults living in the community.^27^

##### Perceived Well-being Outcomes and Use of Health Care Services

Quality of life was measured by the Chinese version of the Short Form 12-item scale, version 2. The scale was translated, validated, and proved to be reliable for use among older Hong Kong Chinese adults.^28^

Depression was measured by the Chinese version of the Geriatric Depression Scale,^29^ which had 15 questions that were used to explore participants’ feelings, with dichotomous answers. The highest possible total score was 15, with higher scores representing more severe depressive symptoms. A cutoff point of 5 showed a sensitivity of 89% and a specificity of 77%.^29^ The number of visits to outpatient clinics, private general practitioners, emergency departments, and hospitals were collected from the participants’ subjective reports.

#### Participant Characteristics

Participant characteristics at baseline were reported for descriptive purposes. They were age, sex, marital status, educational level, work status, financial status, accommodation type, family living in the same household, and caretaking support.

### Statistical Analysis

#### Sample Size

Sample size was calculated based on the *t* test of the primary outcome, which was the difference in mean self-efficacy scores of the 2 groups after the intervention. Assuming a 2-tailed α of .05, statistical power of 0.80, attrition rate of 0.15, and a standardized effect size of 0.45 from a previous self-management interventional study,^30^ 84 participants were required per group. Because this was a pilot study, a target of 35% of the main planned trial was set.^31,32^ As we assume a 15% dropout rate, the total number of participants required was 68.

#### Data Processing and Analysis

Counts and percentages, means and SDs, or medians with minimum and maximum values were presented for categorical or continuous variables when appropriate. Between-group differences before and after the intervention were evaluated using a 2-sided *t* test or Mann-Whitney test.

The between-group (group), within-group (time), and interaction (group × time) effects on the outcome variables were analyzed using a generalized estimating equation. An unstructured working correlation matrix was adopted to indicate the same spacing between measurements for each participant. The generalized estimating equation model included group, time, and interaction of group and time as factors to evaluate each outcome measure except when there was no case at a certain time in a certain group, the outcome would be excluded from being analyzed. The parameter estimate (β), its 95% CI, and the *P* value of each variable were reported. Intention-to-treat was used as the primary analysis. All tests of significance were 2-sided and set at *P* < .05.

## Results

### Baseline Characteristics

Among the 68 participants (mean [SD] age, 71.8 [6.1] years), 12 (17.6%) were men and 56 (82.4%) were women (Table 1). The groups did not have statistically significant differences in either participant characteristics or outcome measures (Table 2) at baseline. The Figure shows the CONSORT diagram.

**Table 2.** Baseline and Postintervention Outcomes in Both Groups

Variable	Mean (SD)			
	Before intervention		After intervention	
	Control (n = 34)	Intervention (n = 34)	Control (n = 34)	Intervention (n = 34)
Self-efficacy score (scale, 10-40)	25.4 (6.7)	27.6 (6.1)	27.1 (5.7)	30.3 (6.3)
Activities of daily living score (scale, 0-100)	93.1 (2.4)	92.6 (2.1)	99.6 (1.7)	99.6 (1.5)
Instrumental activities of daily living score (scale, 0-27)	23.0 (1.9)	22.0 (2.2)	26.1 (1.6)	25.9 (1.5)
Medication adherence score (scale, 12-48)	15.2 (2.4)	16.0 (3.2)	20.9 (13.7)	13.4 (1.9)
Medication taking score (scale, 8-32)	9.94 (1.6)	10.3 (2.2)	14.2 (9.1)	9.1 (1.4)
Prescription refill score (scale, 4-16)	5.2 (1.5)	5.7 (1.8)	6.7 (4.7)	4.3 (0.5)
Quality of life				
PCS (scale, 0-100)	42.5 (8.8)	45.0 (47.5)	42.7 (9.3)	50.1 (7.8)
MCS (scale, 0-100)	50.0 (7.6)	53.1 (8.5)	55.1 (9.7)	58.8 (6.1)
Depression score (scale, 0-15)	2.8 (2.9)	2.1 (2.3)	2.2 (2.9)	1.7 (2.1)
Health care service utilization, No. of times				
Outpatient clinic visits	0.3 (0.8)	0.10 (0.6)	0.1 (0.3)	0.1 (0.5)
Private GP visits	0.4 (1.1)	0.2 (0.6)	0.4 (1.0)	0.3 (0.8)
Emergency department visits	0.0	0.1 (0.2)	0.1 (0.2)	0.0 (0.2)
Hospital admissions	0.1 (0.2)	0.0 (0.2)	0.0 (0)	0.0 (0.2)

Abbreviations: GP, general practitioner; MCS, mental component score; PCS, physical component score.

**Figure.**
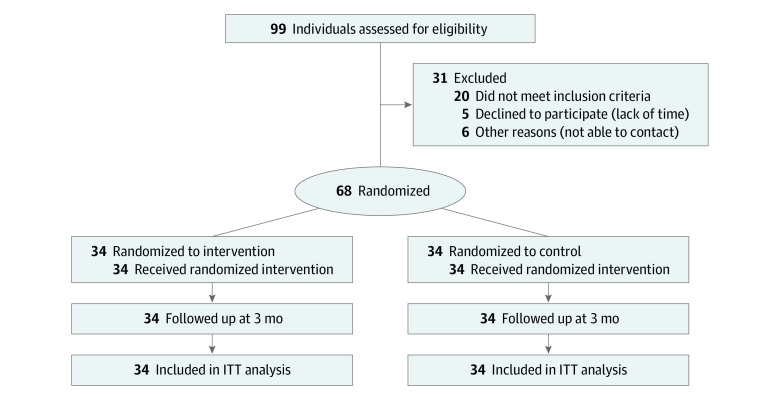
CONSORT Diagram ITT indicates intention-to-treat.

### Treatment Effects

#### Self-efficacy

At 3 months, there was no statistical difference between the groups with respect to change in self-efficacy. Scores for both groups improved from T1 to T2 (β = 1.68; 95% CI, −0.68 to 4.03; *P* = .16) (Table 3).

**Table 3.** Intervention Effects

Variable	Model	
	β (95% CI)	*P* value
Self-efficacy		
Group^a^	2.18 (−0.81 to 5.16)	.15
Time^b^	1.68 (−0.68 to 4.03)	.16
Group × time^c^	1.06 (−2.38 to 4.49)	.55
Basic ADLs		
Group	−0.18 (−1.22 to 0.87)	.74
Time	6.97 (6.08 to 7.87)	<.001^d^
Group × time	0.21 (−0.80 to 1.21)	.69
Instrumental ADLs		
Group	−1.50 (−3.51 to 0.51)	.14
Time	2.62 (1.27 to 3.97)	<.001^d^
Group × time	1.27 (−0.87 to 3.40)	.25
Medication adherence		
Group	0.88 (−0.57 to 2.32)	.23
Time	5.71 (1.00 to 10.42)	.02^d^
Group × time	−8.30 (−13.14 to −3.47)	.001^d^
**Quality of life**		
Physical component score		
Group	2.45 (−1.72 to 6.61)	.25
Time	0.18 (−2.32 to 2.68)	.89
Group × time	4.99 (0.29 to 9.69)	.04^d^
Mental component score		
Group	3.13 (−0.64 to 6.90)	.10
Time	5.16 (1.76 to 8.57)	.003^d^
Group × time	0.57 (−3.52 to 5.06)	.80
Depression		
Group	−0.71 (−1.94 0.53)	.26
Time	−0.59 (−1.58 to 0.42)	.25
Group × time	0.27 (−1.01 to 1.54)	.68
Outpatient clinic visits		
Group	−0.78 (−2.29 to 0.71)	.30
Time	−1.71 (−3.86 to 0.45)	.12
Group × time	1.19 (−1.99 to 4.38)	.46
Private GP visits		
Group	−0.49 (−1.72 to 0.75)	.44
Time	0.00 (−1.35 to 1.35)	>.99
Group × time	0.22 (−1.53 to 1.97)	.80

Abbreviations: ADLs, activities of daily living; GP, general practitioner.

^a^ Group = intervention (reference: control).

^b^ Time = after intervention (reference: before intervention).

^c^ Group × time = intervention × postintervention (reference: control × preintervention, control × postintervention, and intervention × preintervention).

^d^ *P* < .05.

#### ADLs and IADLs

When compared with T1, the ADL scores in both groups increased over time (β = 6.97; 95% CI, 6.08-7.87; *P* < .001) (Table 3). Statistically significant time effects were also found in the IADL scores between T1 and T2 (β = 2.62; 95% CI, 1.27-3.97; *P* < .001).

#### Medication Adherence and QOL

The generalized estimating equation model showed statistically significant between-time interaction effects (β = 5.71; 95% CI, 1.00-10.42; *P* = .02) and group × time interaction effects (β = −8.30; 95% CI, −13.14 to −3.47; *P* = .001), with the telecare group having better scores for medication adherence (Table 3). A statistically significant time effect was discovered for the mental component of the QOL score (β = 5.16; 95% CI, 1.76-8.57; *P* = .003) but not for the physical component of the QOL score. However, there was a statistically significant group × time interaction (β = 4.99; 95% CI, 0.29-9.69; *P* = .04) for the physical component of the QOL score.

#### Depression and Use of Health Care Services

Participants in both groups improved in their depression level in T2 compared with T1; however, there was no statistically significant difference in group, time, or group × time interaction effects (Table 3). In addition, no between-group, within-group, or group × time interaction effects were found in health care service utilization.

## Discussion

To our knowledge, this is the first trial to investigate the effectiveness of a telecare case management program among older adults who are homebound. Although there was no statistical difference between the groups with respect to improvement in self-efficacy, the evidence generated in this randomized clinical trial suggests that an NCM adopting a health-social approach is effective in improving medication adherence and QOL.

The findings of this study are important given that older adults who are homebound are more vulnerable, with restricted access to support services. Older adults who are homebound, like many other older adults, would prefer to age at home.^33^ The current study supported older adults who are homebound in optimizing their capacities to be their own best resources. The support of the NCM and the health-social team encouraged the older adults who were homebound to participate actively in their own care while also allowing them a sense of control over their life.

Given the challenges that COVID-19 brings to home-based programs, the adoption of telecare is a promising alternative care delivery strategy to home visits and may become a new normal practice beyond the pandemic.^34^ However, this new technology is not always welcomed by health care professionals and particularly older adults owing to their lesser proficiency in using technology,^35,36^ which might hinder scaling up and sustaining widespread use of telecare in the long run.^37^ One of the strengths of this program is that, before its commencement, both the older adults who were homebound and the health-social team had received technological training from the research team on how to download and use the WhatsApp function to send, receive, and watch video clips from the smartphone and to troubleshoot technical issues. To ensure that no technical issues would occur, a trial video clip was sent from a research team member to the telecare group participants one day before the first telephone call by the NCM. With all of these resources and supports in place, the older adults who were homebound and enrolled in this study could use the telecare services and build strong connections with the health-social team without leaving their homes. In addition, technological training can also help build the confidence of elderly individuals who are homebound in using a smartphone to receive self-care tips and information, which subsequently may improve their motivation to adhere to the program and maintain their self-care behavior.^38^

Another special feature of the current program was that the team chose video clips rather than oral commands or written materials as a modality for health education and self-care training. Watching videos was found to be the most commonly used and successful strategy to facilitate behavioral change and retain knowledge among older adults.^39^ Evidence has suggested that video outperforms images or written words because the latter cannot convey dynamic body language and facial expressions.^40^ Some studies also concurred that integrating a mobile instruction video into the intervention components of a health care program has shown positive results in terms of promoting a healthy diet,^41^ physical activity,^42^ and smoking cessation,^43^ suggesting that using the video format approach as a platform for promoting self-care might be a viable way forward.

A study by Orgeta et al^44^ revealed that obtaining social support from a telephone call can help older adults to cope with difficulties. It is thus reasonable to surmise that the increase in self-efficacy and QOL and decrease in depression level in the control group participants were owing to the social effects brought about by the monthly social calls.

### Limitations

There are several limitations when interpreting the findings of this study. First, the definition of *homebound* in this study referred to the frequency of leaving the house within the last 6 months. Since pandemic-related social restriction was enforced in Hong Kong during the study period, more older adults, regardless of their physical and functional abilities, preferred to stay in their homes; therefore, there was a chance for older adults without mobility limitations to enroll in the study. Second, the outcomes of this study relied on self-reporting measures by the participants, which might be subject to personal interpretation. Third, this study reported only the outcomes, and there was no information on the process of health-social team coordination of care. Fourth, this was a pilot study with a small number of enrolled participants, which could have limited the statistical power to detect the significance of a group × time interaction effect in the primary and secondary outcome measures.

## Conclusions

Despite its small pilot design with limited generalizability and the absence of a statistical difference between the 2 groups studied, this randomized clinical study suggests that telecare case management may be useful for enabling older adults who are homebound not only to keep a close connection with health care professionals without leaving their home but also to maintain medication adherence and quality of life during the COVID-19 pandemic. A future large-scale study is needed to confirm this result.

## Notes

Supplement 1.
Trial Protocol

Supplement 2.
Data Sharing Statement
