# Supplementary material for: Effect of a Telecare Case Management Program for Older Adults Who Are Homebound During the COVID-19 Pandemic: A Pilot Randomized Clinical Trial
Source: JAMA Netw Open. 2021 Sep 9;4(9):e2123453. doi: 10.1001/jamanetworkopen.2021.23453 (PMC8430449; doi:10.1001/jamanetworkopen.2021.23453)
Supplement: Supplement 1. — Trial Protocol [file jamanetwopen-e2123453-s001.pdf]

Application for NICHE Research Grant

Proposed Research Project

1. Research Title:

Effects of a video-based mHealth program for the homebound older adults: A pilot randomized controlled trial.

2. Relevance to NICHE vision and mission

Homebound older adults are among the highest users in health care systems. The high use of health care services is related to their physical limitations compounded by complex health and social care needs. When the health care system lacks the necessary structure to effectively and efficiently support this population in the community, the use of health care services tend to be higher. Homebound older adults can be difficult to reach because they seldom participate and engage in community services due to their functional limitations, which leaves their health needs largely unrecognized at an earlier stage. This study tries to bring this group of older adults to the providers' attention and design a health-social oriented self-care mHealth program and subject it to empirical testing. The aim of this study is shift the current reactive, cure-oriented approach to a preventive and health promoting model, empowering the homebound older adults to take an active role in their health, be responsive to their care needs, and subsequently improve their holistic health. The main concept of this study is thus consistent with the vision and mission of NICHE, which is to bring holistic health to mankind through promoting the awareness of holistic health and practice of holistic health care.

3. Background:

The number of homebound older adults has been rising rapidly due to the ever-increasing growth of aging population. The latest report showed that approximately 2 million older adults aged 65 and older in the United States are considered as homebound [1]. Similar to the figures of several OECD countries (i.e. 5%-20%) [2], the prevalence rate of homebound older adults in Hong Kong has been estimated at about 8.9% of the population [3], with some estimating the percentage to be as high as 12.5% [4]. Homebound older adults are defined as those who confined to their home due to a combination of chronic conditions and physical and functional impairments, and normally not able to go outdoors more than once per week [5]. Their homebound state is considered as a barrier to gain access to the community and restrict from joining community programs. When these older adults experience health and social problems, many of them struggle to manage on their own and rely heavily on acute and tertiary care if no other help is available [2]. A statistical report revealed that homebound older adults had significantly higher rate of emergency admission use and were more likely to admit to hospital and long-term care facilities and consumed more health care expenditures when compared to their non-homebound counterparts [6]. The high use of health care services is not only related to their physical limitations and complex health and social care needs, but also reflective of the lack of a comprehensive primary health care structure to support the self-care of this population in the community [7].
In response to the need of those homebound older adults who live in old private housing, the Hong Kong Red Cross initiates a community health care project that includes health checks, social activities, health education, and emotional support since 2013 [8]. This program aims to enhance the quality of life and improve health condition of homebound

older adults, as well as arouse the interest of other community organizations to pay more attention to these vulnerable, but usually neglected group of people. Since then, District Elderly Community Centre (DECC) and Neighbourhood Elderly Centre (NEC) also provide a range of outreach services such as meal and laundry services, transportation, health assessment, and provision of information on community resources to enable homebound older adults remain living independently in their own homes [9]. These services, though effective, encompass the problem of fragmentation and diversity in objectives which may not optimally meet the holistic needs of the older adults.

In 2012, a program called Integrated Care and Discharge Support for elderly patients (ICDS) has been launched in Hospital Authority to address the difficulties that discharged elderly patients encounter when they return home. The program targeted at frail elderly patients who are discharged from medical ward and at high risk for functional decline and readmission [10]. For those older adults who are qualified for this program, a health care team that includes geriatricians, nurses, occupational therapists, and physiotherapists work together and provide pre-discharge planning, post-discharge rehabilitation service, home safety assessment and modification, and counselling through home visit and telephone follow-up in an effort to enhance continuation of support in the community. While preliminary results showed that the program has successfully reduced acute hospital admissions, hospital bed days and Accident and Emergency department (AED) attendance episodes [10, 11], the ICDS program has a few limitations. Firstly, it is hospital-based which service has theoretically focused on curative rather than maintenance aspects of care. In the context of limited medical resources, it seems impractical to provide consistent and sustainable care to discharged homebound older adults primarily by hospitals. In the long run, health care has to shift the paradigm from hospital-based, treatment-centred practice to a community-based, more prevention focused model in order to shuffle off the growing burden of aging population [12]. Besides, since this is a hospital-based program, it can only reach a fraction of those who were admitted to the hospital, leaving the majority living independently in the community unattended at an earlier stage before they become frequent hospital user. Secondly, it does not facilitate the integration of patient perspectives in care decisions related to their day-to-day living because the hospital-based program is initiated by a illness-related reason and the care planning is designed based on a medical model with limited patient involvement. These shortcomings suggest that research adopting a community-based model is needed to build evidence to improve the care for homebound older adults in Hong Kong.

Although homebound older adults have higher levels of comorbidity and physical impairments than their non-homebound cohorts do, they have shown the ability to take good care of themselves at home when they could be given opportunities to learn about their health conditions. A feasibility study showed that homebound older adults were able to access health information on the internet and managed their chronic diseases at home after attending computer-training lessons [13]. Another pilot study demonstrated that homebound older adults could successfully manage and adhere to their medicines when they were offered a self-care agenda by pharmacists [14]. Evidence suggested that people who are capable to adhere to self-care activities show a decrease in health services utilization and the healthcare cost, with increase in satisfaction level, sense of control, well-being, self-efficacy, quality of life, and most importantly, stay independently in their own community as long as possible [15, 16, 17]. Yet, to date, there are few programs attempted to empower the homebound older adults to take an active role in their health.

Community health care organizations are frequently under economic constraints to provide better services with fewer resources [18]. Mobile health (mHealth) is emerging

as a solution for overcoming this challenge and offering low cost, ready access, and individualized care to people [19]. mHealth not only enables information sharing across professional and organizational boundaries, it provides an easy-to-use platform for connecting older adults electronically with the health care professionals without the need of leaving home [20]. According to WHO, mHealth is defined as a medical and public health practice using the core utility of mobile and wireless technologies to support the achievement of health objectives [21]. A systematic review found that mobile instruction video is the most commonly used and successful strategy in mHealth programs to facilitate behavioral change, increase knowledge and understanding, and improve compliance with medical instructions for older adults [22]. Evidence showed that video have outperformed images or written words since they cannot convey dynamic body language and facial expressions [23]. This delivery channel proves to be particularly useful in older adults who have limited education, motivation and health literacy as video requires less reading of multiple pages of written material [24]. Recent studies integrating mobile instruction video in primary health services have demonstrated positive results on promoting physical activity [25], balanced diet intake [26], and medication adherence [27], which suggested that employing the video-format approach as a platform for promoting self-care might be a viable way forward.

Existing studies of mHealth interventions have demonstrated inconsistent results on improving self-care of older adults. A trial involved one-way video messages in a diabetes care program showed that there was a larger rate of decline in blood glucose in older adults who received the messages than those who received no messages, but the difference was not statistically significant [28]. Another study provided basic program information, video and a simple fact sheet about heart failure to a group of older adults who have heart failure with the aim of improving their self-care ability [29]. The results indicated that there were no significant differences in physical activity, health status, and health care cost and utilization between intervention and control groups. In fact, homebound older adults are a group of disabled person who have multiple health and social needs, mHealth intervention that provided self-care information alone may not be enough for them to remain living in the community. In order to promote their self-care and independent living, building a health-social team in the community that can provide individualized care is imperative. Within the team, nurse play an important role in delivering and coordinating most of the health care services including continuous comprehensive assessment, case management, health education, and medical and social referral; while social worker is well-positioned to mobilize tangible community resources, and provide psychological and financial reassurance. To our knowledge, there is no investigating the use of health-social team with the support of mHealth on empowering and promoting self-care of homebound older adults. This proposal therefore endeavors to develop a mHealth program, with the integrated efforts of a health-social team, on increasing self-care ability and health among homebound older adults and submit it to empirical testing. This study adds to the knowledge gap in using mHealth supported by a health-social team in enhancing quality of life and self-care and meeting the needs of these particularly vulnerable older adults.

4. Originality and Impact:

Homebound older adults are an understudied population that often lives with multiple chronic diseases. Without regular access to primary preventive care, these frail older adults have high risk of moving into long-term care facilities rather than staying in the community. As for the older adults themselves, they would prefer living independently in their own homes rather than receive institutional care due to their emotional

attachment to their familiar and comfortable environment. Currently, the health and social services provided to the homebound older adults in Hong Kong are both manpower-intensive and episodic in nature. This study aims to facilitate age in place by providing holistic care to these vulnerable, but usually neglected group of people with the support of technology and a health-social team in the community. To our knowledge, this program is one of the few to not only include the homebound older adults who are traditionally excluded from the benefits of mHealth, but also empower them to take care of themselves so as to increase their self-efficacy, quality of life, maintain basic and instrumental activities of daily living, and reduce unnecessary hospital admission and expenditure. It employs an approach that addresses the health and social needs of client in a comprehensive way including environmental, psychosocial, physiological, and health-related behaviors. We believe that these factors are inter-related and the successful management of one aspect may enhance the impact of another. For example, motivating the clients to observe healthy behavior and medication adherence can improve health status, thus quality of life.

5. Research Questions, Aims and Hypotheses:

Aim:

The aim of this study is to test the effects of a video-based mHealth program and the control group receiving usual care on health outcomes, perceived well-being and health service utilisation.

Research questions:

1. Is there a difference in health outcomes (i.e. activities of daily living, instrumental activities of daily living, medication adherence) between the intervention group and the control group?
2. Is there a difference in perceived well-being outcomes (i.e. quality of life, self-efficacy, depression) between the intervention group and the control group?
3. Is there a difference in health service utilisation outcomes (i.e. outpatient clinic, emergency room admission, hospital admission) between the intervention group and the control group?

Hypothesis:

There is no difference in health outcomes, perceived well-being and health service utilisation between the subjects receiving video-based mHealth program and receiving usual care.

6. Subjects and Study Methodology:

Study design:

This is a randomized controlled trial.

Setting and subjects:

This study is supported by five community centres run by the Lutheran group. The five community centres are widely scattered throughout Hong Kong island, Kowloon, and New Territories, which help maximize the generalizability effect of this program.

The leaders of the community centres will help draw a list of potential subjects. Simple random sampling with fixed sample size will be employed to recruit subjects. The inclusion criteria of this study include: (1) people who are aged 60 or above, (2) go outdoor less than once per week in current 6 months [14], (3) living within the service area of community centre, and (4) using smartphone. The exclusion criteria are: (1) diagnosed with dementia, (2) unable to hear, see or communicate, (3) bed-bound, (4) active psychiatric illness with recent hospital admission within last 6 months, (5) living within an area with no internet coverage, and (6) already engaged in other mHealth

programs. The potential subjects will be approached and invited to participate in the study. Consent form will be signed once the subject agrees to participate. The random assignment schedule, generated using the computer software Research Randomizer, will be compiled by a research team member who is not involved in subject recruitment. The group assignments will be put in a sealed envelope and revealed sequentially at the time of randomization. The research assistant, after successfully recruited a subject, will call the research team member for the random assignment. The research team member who has no knowledge of the identity of the subject will make the assignment based on the computer number ('1' = video-based mHealth program; '2' = control group). In this study, the research assistant who collects the data is blinded but the subjects and the health care providers who involve in the intervention are not.

Sample size

The sample size calculation is based on power analysis. Assuming a two-tailed alpha of 0.05, a probability of 0.2 for beta error (80% power), and an effect size of 0.436 after taking reference of previous video-based mHealth programs with the same primary outcome measure (self-efficacy) [30]. Eighty-four per group are required. With reference to the 10% to 15% attrition rate reported in the previous programs [31, 32], we assume a 15% drop-out rate in this study, thus the total sample size needed is 97 participants per group, i.e. a total of 194 participants. However, since this is a pilot study, according to a study [33], at least 9% of the sample size of the main planned trial should be used. To ensure the power is enough to estimate the group difference, 35% of the main planned trial is set [34]. The total number of clients required therefore is 68.

Interventions

A 3-month program will be designed in this study. There will be two groups involved in this study with description as follows.

<u>Video-based mHealth program</u>

This group of participants will receive a video-based mHealth program which includes two main elements: 1) nurse case management supported by a social service team, 2) individual-specific video messages covering self-care topics delivered via smartphone. Upon enrolment, each client in the intervention group is assigned to a nurse case manager. The nurse case manager will conduct a structured assessment by using the Omaha System via telephone. The Omaha System is a comprehensive assessment-intervention-evaluation tool that identifies 42 problems under the domains of environmental, psychological, physiological, and health-related behaviours. The Omaha System has been found to be applicable for older adults in the community and proven valid to be used in Hong Kong [30]. Following assessment, nurse case manager will guide and empower the clients to set their own goals and co-decide their own plans to manage their health problems.

Apart from monitoring the progress of the clients in accordance to the contract goals in a weekly telephone call, the nurse case manager will also send weekly, individual-specific videos of tips and reminders via smartphone. These videos cover topic of importance to clients according to the result of the Omaha System. The content of videos includes but not limits to chronic disease self-management, healthy behaviours, simple self-care practices, and medication knowledge. The selected videos are limited to ten minutes to accommodate the short attention span of older adults [35]. All videos that provided to the intervention group are originating from reliable sources such as Hospital Authority, Department of Health or credible health or charity organizations. Nurse case manager will first send a testing video to the client for the first time to ensure that the client can receive the video on his/ her mobile. The client can either download the videos or view online. The videos will run continuously unless the client presses the "pause" button. Videos can be replayed once clicking the "play" button again.

A protocol for referral system will be established with the full support of health-social team in accordance with the conditions and the needs of the client. The nurse can initiate client referral according to the set guidelines. The health referrals include primary care consultation and hospital service if indicated. Social referrals include home meal service, psychological counselling and the like. In this team, the nurse case manager is responsible to provide health-related services such as health education and drug compliance monitoring, while social workers can provide services including arrange home-delivered meals, provide psychosocial support, and mobilize community resources available in the district. A monthly case conference will be held in the community centre between nurse case manager and social worker. Issues such as progress and concern of the clients, suggestions for interventions, and requirement of modification or adjustment of goals will be discussed during the meeting. The shared responsibilities are based on standardized protocols and agreed referral forms and records.

Control group

Both the intervention and control groups will receive their respective community services as usual. The most common services provided by community centres include meal and laundry services, transportation service, health check and education, and social activities, but most of them are episodic in nature. Social calls will be provided to the control group clients by a research team member who will not involve in data collection to minimize possible social effects. The social questions such as "where will you go tomorrow?" and "what is your favourite TV program?" will be set in the protocol. The research team member will strictly follow the protocol and will not provide health advice. The clients will be asked to seek medical help whenever the older adults express concern about their health. The flow of the intervention program can be found in supplementary figure 1.

7. Outcomes and Data Analysis:

Data will be collected at two time points—pre-intervention (T1) and post-intervention (T2). The baseline and the post-intervention data will be collected by the research assistant who is blinded to the grouping. The research assistant will be trained and tested on the inter-rater and intra-rater reliability. Five percent of the data will be randomly selected for independent review to ensure data quality.

Outcome measures

There are 4 sets of measures: namely demographics, health, perceived well-being and health service utilisation outcomes.

Background demographic data

The background demographic data that collected at baseline include age, gender, education level, marital status, years of using smartphone, work status, accommodation type, financial status, family living in the same household, and caretaking support. The entire set of baseline measures has been validated and its reliability confirmed in previous study [35].

Health outcomes (activities of daily living, instrumental activities of daily living, medication adherence)

Activities of daily living will be measured by the Chinese version of Barthel index. This 20-item questionnaire was validated with a reported inter-rater reliability of 0.99 [36]. Instrumental activity of daily living will be measured by the Chinese version of Lawton Instrumental activity of daily living [37]. The scale covers tasks such as telephone, shopping, meal, and transport. The Cronbach's alpha internal consistency and the inter-rater reliability was 0.86 and 0.99 respectively [37].

Medication adherence will be measured using the Adherence to Refills and Medication

Scale [38]. The 12-item self-reporting questionnaire was designed to assess the respondent's ability to take and refill all prescribed medications under different circumstances. It has been shown to be highly valid in identifying medication adherence issues of community-dwelling older adults [38].

Perceived well-being outcomes (quality of life, self-efficacy, depression)

Quality of life will be measured by SF-12v2 which has been translated, validated and proven reliable for use among the Hong Kong Chinese population. The internal consistency and test-retest reliabilities were good, and the SF-12v2 summary scores explained more than 80% of the total variances of the SF-36v2 summary scores [39].

Self-efficacy will be measured by General Self-Efficacy Scale. This scale is used to determine how people judge their ability to handle difficult situations or solve their own problems, and thus can be a way to assess the effectiveness of empowerment programs. The scale has been validated with high Cronbach's alpha internal consistency (0.89) [40].

Depression will be measured by the Chinese version of the Geriatric Depression Scale [41]. Good validity and reliability were reported in this scale, with criterion-related validity 0.95 and test-retest reliability 0.85 among the elderly Chinese population [42].

Health service utilisation outcomes (outpatient clinic, emergency room admission, hospital admission)

Health service utilisation will be measured by the number of attendances to government out-patient clinic, and number of unscheduled visits to the emergency department and hospital admission.

Data analysis

Descriptive analyses will be used for describing the data collected in the baseline such as background demographic data. The baseline descriptors will be presented as mean and standard deviation for continuous variables, median and quartile range when the continuous variables are not normally distributed, and percentage and frequency for categorical variables. The intervention group and the control group will be compared on all demographic data to ensure the randomness of the group assignment and to identify possible need of adjustment in further analysis. Poisson regression will be used to examine the mean difference of health service utilisation outcomes. Generalized Estimating Equation will be used to compare any differences in health and perceived well-being outcomes between groups. The Chi-squared test or Fisher's exact test will be used to compare any differences in background demographic data and the three sets of outcomes. Intention-to-treat (ITT) will be used as the primary analysis for missing data in this study.

8. Ethical and Safety Considerations:

This study will not cause any discomfort or painful sensation to participants. Information about and an explanation of the ethical observations of the study will be provided to all eligible participants and they will be asked to sign an informed consent. Participants will be reassured that they can withdraw from the study any time without any adverse consequences. They will remain anonymous and all data will be identified by a case number only. All data collected from questionnaires will be stored in a cabinet that required password to open. The results of this study may be used in future research and for publication. Ethical approval will also be sought from the Human Subject Sub-committee of the Hong Kong Polytechnic University.

Reference

1. Xiang, X., Chen, J., & Kim, M. (2019). Trajectories o homebound status in Medicare Beneficiaries aged 65 and older. *The Gerontologist*, *XX*, 1-11.
2. Kim, C.O., & Jang, S.N. (2018). Home-based primary care for homebound older adults: Literature review. *Annals of Geriatric Medicine and Research*, *22*(2), 62-72.
3. Yu, R., Wong, M., Chang, B., Lai, X., Lum, C.M., Auyeung, T.W., ... Woo, J. (2016). Trends in activities of daily living disability in a large sample of community-dwelling Chinese older adults in Hong Kong: An age-period-cohort analysis. *BMJ Open*, *6*(12), e013259.
4. Woo, J., Yu, R., Wong, M., Yeung, F., Wong, M., & Lum, C. (2015). Frailty screening in the community using the FRAIL scale. *Journal of the American Medical Directors Association*, *16*(5), 412-419.
5. Sterling-Fox, C. (2019). Access to five nonprimary health care services by homebound older adults: An integrative review. *Home Health Care Management & Practice*, *31*(1), 55-69.
6. Shafir, A., Garrigues, S.K., Schenker, Y., Leff, B., Neil, J., & Ritchie, C. (2016). Homebound patient and caregiver perceptions of quality of care in home-based primary care: A qualitative study. *Journal of the American Geriatrics Society*, *64*, 1622-1627.
7. Gray, C.S., Barnsley, J., Gagnon, D., Belzile, L., Kenealy, T., Shaw, J., … Wodchis, W.P. (2018). Using information communication technology in moels of integrated community-based primary health care: Learning from the iCOACH case studies. *Implementation Science*, *13*, 87.
8. Hong Kong Red Cross. (2013). *Red Sham Project*. Retrieved from https://www.redcross.org.hk/en/community_healthcare/comcareproject/introduction.html on 22 May, 2019.
9. Hong Kong Policy Research Institute. (2017). *Aging in Place—Family and community support and Care*. Retrieved from http://www.hkpri.org.hk/en/research-detail/aging-in-place on 22 May, 2019.
10. Lin, F.O.Y., Luk, J.K.H., Chan, T.C., Mok, W.W.Y., & Chan, F.H.W. (2015). Effectiveness of a discharge planning and community support programme in preventing readmission of high-risk older patients. *Hong Kong Medical Journal*, *21*, 208-216.
11. Chan, S., Kwong, P., Kong, B., To, K., Ho, J., & Wong, C.P. (2008). Improving health of high risk elderly in the community—The HARPRE. In *Symposium on Community Engagement, creating a synergy for community health*, Hong Kong East Cluster, Hospital Authority, Hong Kong.
12. van Velsen, L., Illario, M., Jansen-Kosterink, S., Crola, C., di Somma, C., Colao, A., & Vollenbroek-Hutten, M. (2015). A community-based, technology-supported health service for detecting and preventing frailty among older adults: A participatory design development process. *Journal of Aging Research*, *2015*, 1-9.
13. Choi, N.G., & Garcia, A. (2014). A feasibility study of low-income homebound older adults' participation in an online chronic disease self-management program. *Home Health Care Services Quarterly*, *33*, 106-120.
14. Latif, A., Mandane, B., Anderson, E., Barraclough, C., & Travis, S. (2018). Optimizing medicine use for people who are homebound: An evaluation of a pilot domiciliary medicine use review service in England. *Integrated Pharmacy Research and Practice*, *7*, 33-40.
15. Lupon, J., Gonzalez, B., Urrutia, A., Arenas, M., Domingo, M., Altimir, S., & Valle, V. (2008). Patient's self-care improvement with nurse education intervention in Spain assessed by the European heart failure self-care behavior scale. *European Journal of Cardiovascular Nursing*, *7*, 16-20.
16. Richard, A.A., & Shea, K. (2011). Delineation of self-care and associated concepts. *Journal of Nursing Scholarship, 43*(3), 255-264.
17. Van der Wal, M.H., & Jaarsma, T. (2008). Adherence in heart failure in the elderly: Problem and possible solutions. *International Journal of Cardiology, 125*, 203-220.
18. Gellis, Z.D., Kenaley, B., McGinty, J., Bardelli, E., Davitt, J., & Have, T.T. (2012). Outcomes of a telehealth intervention for homebound older adults with heart or chronic respiratory failure: A randomized controlled trial. *The Gerontologist*, *52*(4), 541-552.

19. Berg, T., Winterton, R., Petersen, M., & Warburton, J. (2017). 'Although we're isolated, we're not really isolated': The value of information and communication technology for older people in rural Australia. *Australasian Journal on Ageing*, *36*(4), 313-317.
20. Yao, N., Ritchie, C., Cornwell, T., & Leff, B. (2018). Use of home-based medical care and disparities. *Journal of the American Geriatrics Society*, *66*(9), 1716-1720.
21. World Health Organization. (2011). *mHealth: New horizons for health through mobile technologies: Second global survey on eHealth*. Geneva, Switzerland: WHO Global Observatory for eHealth.
22. Free, C., Philips, G., Galli, L., Watson, L., Felix, L., Edwards, P., … Haines, A. (2013). The effectiveness of mobile-health technology-based health behaviour change or disease management interventions for health care consumers: A systematic review. *Plos Medicine*, *10*(1), e1001362.
23. Schooley, B., Nicholas-Rocca, T.S., & Burkhard, R. (2015). Patient-provider communications in outpatient clinic settings: A clinic-based evaluation of mobile device and multimedia mediated communications for patient education. *JMIR mHealth and uHealth*, *3*(1), e2.
24. Ben-Zeev, D., Brian, R.M., Aschbrenner, K.A., Jonathan, G., & Steingard, S. (2018). Video-based mobile health interventions for people with schizophrenia: Bringing the "Pocket therapist" to life. *Psychiatric Rehabilitation Journal*, *41*(1), 39-45.
25. Direito, A., Carraca, E., Rawstorn, J., Whittaker, R., Maddison, R., & Carraca, E. (2017). mHealth technologies to influence physical activity and sedentary behaviors: Behavior change techniques, systematic review and meta-analysis of randomized controlled trials. *Annals of Behavioral Medicine*, *51*(2), 226-239.
26. Spring, B., Pellegrini, C., McFadden, H.G., Pfammatter, A.F., Stump, T.K., Siddique, J., … Hedeker, D. (2018). Multicomponent mHealth intervention for large, sustained change in multiple diet and activity risk behaviors: The make better choices 2 randomized controlled trial. *Journal of Medical Internet Research*, *20*(6), 1-1.
27. Dekoekkoek, T., Given, B., Given, C.W., Ridenour, K., Schueller, M., & Spoelstra, S.L. (2015). mHealth SMS text messaging interventions and to promote medication adherence: An integrative review. *Journal of Clinical Nursing*, *24*(19-20), 2722-2735.
28. Bell. A.M., Fonda, S.J., Walker, S., Schmidt, V., & Vigersky, R.A. (2012). Mobile phone-based video messages for diabetes self-care support. Journal of Diabetes Science and Technology, 6(2), 310-319.
29. Veroff, D.R., Sullivan, L.A., Shoptaw, E.J., Venator, B., Ochoa-Arvelo, T., Baxter, J.R., … Wennberg, D. (2012). Improving self-care for heart failure for seniors: The impact of video and written education and decision aids. *Population Health Management*, *15*(1), 37-45.
30. Chow, S.K.Y., & Wong, F.K.Y. (2014). A randomized controlled trial of a nurse-led case management programme for hospital-discharged older adults with co-morbidities. *Journal of Advanced Nursing*, *70*(10), 2257-2271.
31. McNulty, T.C., & Fisher, A.G. (2013). Comparison of activities of daily living ability of older adults who are homebound and nonhomebound. *Physical & Occupational Therapy in Geriatrics*, *31*(2), 89-102.
32. Laforest, S., Nour, K., Gignac, M.A.M., Gauvin, L., & Parisien, M. (2012). The role of social reinforcement in the maintenance of short-term effects after a self-management intervention for frail housebound seniors with arthritis. *Canadian Journal on Aging*, *31*(2), 195-207.
33. Cocks, K., & Torgerson, D.J. (2013). Sample size calculations for pilot randomized trials: A confidence interval approach. *Journal of Clinical Epidemiology*, *66*, 197-201.
34. Whitehead, A.L., Julious, S.A., Cooper, C.L., & Campbell, M.J. (2016). Estimating the sample size for a pilot randomized trial to minimize the overall trial sample size for the external pilot and main trial for a continuous outcome variable. *Statistical Methods in Medical Research*, *25*(3), 1057-1073.
35. Commodari, E., & Guarnera, M. (2008). Attention and aging. *Aging Clinical and Experimental Research*, 20(6), 578-584.

36. Marek, K.D., Stetzer, F., Ryan, P.A., Bub, L.D., Adams, S.J., Schlidt, A., … O'Brien, A.M. (2013). Nurse care coordination and technology effects on health status of frail older adults via enhanced self-management of medication. *Nursing Research*, *62*(4), 269-278.
37. Leung, S.O.C., Chan, C.C.H., & Shan, S. (2007). Development of a Chinese version of the Modified Barthel Index—validity and reliability. *Clinical Rehabilitation*, *21*, 912-922.
38. Lawton, M.P., & Brody, E.M. (1969). Assessment of older people—self-maintaining and instrumental activities of daily living. *Gerontologist*, *9*, 179-186.
39. Jin, H.K., Kim, Y.H., & Rhie, S.J. (2016). Factors affecting medication adherence in elderly people. *Patient Preference and Adherence*, *2016*(10), 2117-2125.
40. Lam, E.T.P., Lam, C.L.K., Fong, D.Y.T., & Huang, W.W. (2013). Is the SF-12 version 2 health survey a valid and equivalent substitute for the SF-36 version 2 health survey for the Chinese? *Journal of Evaluation in Clinical Practice*, *19*, 200-208.
41. Leung, D.Y.P., & Leung, A.Y.M. (2011). Factor structure and gender invariance of the Chinese General Self-Efficacy scale among soon-to-be-aged adults. *Journal of Advanced Nursing*, *67*(6), 1383-1392.
42. Chi, I., Yip, P.S.F., Chiu, H.F.K., Chou, K.L., Chan, K.S., Kwan, C.W., … Caine, E. (2005). Prevalence of depression and its correlates in Hong Kong's Chinese older adults. *The American Journal of Geriatric Psychiatry*, *13*(5), 409-416.
