# Supplementary material for: Effect of a Telecare Case Management Program for Older Adults Who Are Homebound During the COVID-19 Pandemic: A Pilot Randomized Clinical Trial
Source: JAMA Netw Open. 2021 Sep 9;4(9):e2123453. doi: 10.1001/jamanetworkopen.2021.23453 (PMC8430449; doi:10.1001/jamanetworkopen.2021.23453)
Supplement: Supplement 2. — Data Sharing Statement [file jamanetwopen-e2123453-s002.pdf]

Data Sharing Statement

Wong. Effect of a Telecare Case Management Program for Older Adults Who Are Homebound During the COVID-19 Pandemic. *JAMA Netw Open*. Published September 09, 2021. doi:10.1001/jamanetworkopen.2021.23453

Data
Data available: No
